# Rethinking the physiological cross-sectional area of skeletal muscle reveals the mechanical advantage of pennation

**DOI:** 10.1098/rsos.240037

**Published:** 2024-09-18

**Authors:** Robert Rockenfeller, Michael Günther, Christofer J. Clemente, Taylor J. M. Dick

**Affiliations:** ^1^ Mathematical Institute, University of Koblenz, Koblenz, Germany; ^2^ School of Biomedical Sciences, University of Queensland, Brisbane, Queensland, Australia; ^3^ School of Science, Technology and Engineering, University of the Sunshine Coast, Sippy Downs, Queensland, Australia; ^4^ Computational Biophysics and Biorobotics, Institute for Modelling and Simulation of Biomechanical Systems, University of Stuttgart, Stuttgart, Germany; ^5^ Friedrich–Schiller–Universität, Jena, Germany

**Keywords:** muscle architecture, biomechanics, mathematical modelling, physiology

## Abstract

The shape of skeletal muscle varies remarkably—with important implications for locomotor performance. In many muscles, the fibres are arranged at an angle relative to the tendons’ line of action, termed the pennation angle. These pennate muscles allow more sarcomeres to be packed side by side, enabling the muscle to generate higher maximum forces for a given muscle size. Historically, the physiological cross-sectional area (PCSA) has been used to capture both the size and arrangement of muscle fibres, and is one of the best predictors of a muscles capacity to produce force. However, the anatomical and mechanical implications of PCSA remain ambiguous as misinterpretations have limited our ability to understand the mechanical advantage of pennate muscle designs. We developed geometric models to resolve the mechanistic and functional impacts of pennation angle across a range of muscle shapes and sizes. Comparisons among model predictions and empirical data on human lower limb muscles demonstrated how a pennate arrangement of fibres allows muscles to produce up to six times more isometric force when compared with non-pennate muscles of the same volume. We show that in muscles much longer than thick, an optimal pennation angle exists at which isometric force is maximized. Using empirically informed geometric models we demonstrate the functional significance of a pennate muscle design and provide a new parameter, pennation mechanical advantage, which quantifies this performance improvement.

## 1. Introduction

Skeletal muscle is the engine that powers human and animal locomotion. It produces the force that allows animals to achieve movements ranging from slow and steady to rapid and powerful. This diversity in locomotor function is, in part, enabled through variation in muscle design. Muscle architecture, the arrangement of fibres within a muscle and the organization of muscles within the body, has a significant influence on whole-animal performance. For example, muscles characterized by long parallel fibres are better suited for large excursions and high shortening velocities [1] owing to a greater number of sarcomeres in series. Muscles with a pennate design possess fibres arranged at an angle relative to the muscle’s line of action (tendon), allowing more fibres and thus more sarcomeres to be packed in parallel—a strategy favouring force production [2].

Directly measuring *in vivo* muscle forces remains a fundamental challenge owing to the invasive nature and technical requirements. Physiological cross-sectional area (PCSA, symbol $A_{\text{P}}$ ) is often used as a proxy for a muscle’s maximum force-generating capacity, and can be combined with other properties (e.g. force-length or force-velocity characteristics) to create models of muscle force production [3]. Theoretically, PCSA is defined as the sum of the cross-sectional areas (CSAs) of all the muscle fibres within a muscle. Yet this is challenging to measure *in vivo*, and so PCSA is often considered as the area of the cross-section oriented perpendicular to the muscle fibres, but only if this plane intersects all muscle fibres present in that muscle. In muscles with more complex architectures, for example, the human soleus [4], there is not one single plane that lies both perpendicular to the muscle fibres and intersects all muscle fibres within the muscle. In practice, PCSA is calculated as muscle volume (mass divided by density) divided by optimal fibre length at which maximum isometric force can be exerted, i.e.


(1.1) $$\text{PCSA}:A_{\text{P}}\;\left[{\text{cm}}^{2}\right]=\frac{\text{muscle (belly) mass}\;\left[\text{g}\right]}{\text{mass density}\;\left[{\text{g/cm}}^{3}\right]\cdot \text{optimal fibre length}\;\left[\text{cm}\right]}\;,$$

which can be measured using a variety of different surgical, chemical, weighing and imaging techniques [5]. We show the equivalence of the theoretical and practical definition of PCSA for a variety of different fibre architectures and muscle shapes in appendix A. Note that the definition of ‘optimal’ fibre length is already ambiguous throughout the literature, refer to [6] for a recent review. Herein, we refer to ‘optimal’ fibre length as the average length of all fibres within the geometric configuration at which the muscle can produce maximum force isometrically.

Knowledge about the the pennate arrangement of muscle fibres dates back to over 350 years ago [7–9]. As recently as 40 years ago, one of the earliest studies to explore the relationship between muscle architecture and function was performed on isolated muscles in guinea pig [10]. Powell *et al.* measured muscle (belly) mass, fibre length, pennation angle and maximum isometric forces in 11 hindlimb muscles. They developed a simple model that predicted maximum specific tension $P_{0}$ from architecture, including the pennation angle and termed it *functional* cross-sectional area (FCSA, symbol $A_{\text{F}}$ ) in the abstract. They demonstrated that FCSA was able to predict the measured values of maximum isometric force almost perfectly ( r=0.99 ):


(1.2) $$\begin{array}{ll} \text{FCSA}:A_{\text{F}}\;\left[{\text{cm}}^{2}\right] & =\frac{\text{muscle (belly) mass}\;\left[\text{g}\right]\cdot cos\;\left(\text{pennation angle}\right)\;\left[\;\right]}{\text{mass density}\;\left[{\text{g/cm}}^{3}\right]\cdot \text{optimal fibre length}\;\left[\text{cm}\right]}\\ \;\;\;\; & =A_{\text{P}}\cdot cos\;\left(\text{pennation angle}\right) \end{array}.$$

Thus equation (1.2) provided a framework to better understand the links between muscle CSA, fibre geometry and force-generating capacity. However, some terminology confusion was instigated because these authors used FCSA and PCSA synonymously within their seminal work [10]. For example, in their table 1, values for FCSA rather than PCSA are reported, although they denoted it PCSA. Unfortunately, this confusion in terminology has since been passed on nearly ubiquitously in the human and comparative biomechanics, anatomy and physiology communities for 40 years in both textbooks (see [11, eqn 1.1] for a prominent example) and papers (see [2,5,12–14]), for one representative example per decade)—with only few exceptions (see [15,16]).

**Table 1.** Empirical data used to inform geometric model. Muscle architecture for a range of lower limb muscles from [12, table 3] with their abbreviations, belly masses ( M ), belly lengths ( $\ell_{m}$ ), optimal fibre lengths ( $\ell_{fib}$ ) and pennation angle at rest ( $\alpha_{rest}$ ). The fibre width ( $\tau_{m}$ ) was calculated from belly mass and length and compared with literature values ( $\tau_{m,lit}$ ). Length-to-thickness ratio ( ξ ), ACSA ( $A_{\text{A}}$ , equation (2.1)), GCSA ( $A_{\text{G}}$ ), PCSA ( $A_{\text{P}}$ , equation (2.3)) and FCSA ( $A_{\text{F}}$ , equation (2.4)) were calculated as explained in the text. For comparison, the experimental $A_{\text{P},\exp}$ [12] is listed. PMA ( P , equation (2.5)) was calculated as the ratio between $A_{\text{F}}$ and $A_{\text{A}}$ .

muscle name	abbrev.	M	$\ell_{m}$	$\ell_{fib}$	α	$\tau_{m}$	$\tau_{m,lit}$	ξ	$A_{\text{A}}$	$A_{\text{G}}$	$A_{\text{P}}$	$A_{\text{P},\exp}$	$A_{\text{F}}$	P
		(g)	(cm)	(cm)	(deg)	(cm)	(cm)	()	(cm ${,}^{2}$ )	(cm ${,}^{2}$ )	(cm ${,}^{2}$ )	(cm ${,}^{2}$ )	(cm ${,}^{2}$ )	()
adductor brevis	AB	54.6	15.4	10.3	6.1	2.53	—	6.08	5.04	5.1	5.97	5.03	5.94	1.18
adductor longus	AL	74.7	21.8	10.8	7.1	2.49	—	8.78	4.86	4.93	7.14	6.55	7.09	1.46
adductor magnus	AM	325	37.9	14.4	15.5	3.94	—	9.63	12.2	13.1	33.4	21.3	32.2	2.65
biceps femoris long	BFL	113	34.7	9.76	11.6	2.43	2.04 [44]	14.3	4.64	4.83	14.1	11.5	13.8	2.97
biceps femoris short	BFS	59.8	22.4	11	12.3	2.2	—	10.2	3.79	3.97	9.03	5.22	8.82	2.33
ext. digitorum longus	EDL	41	29	6.93	10.8	1.6	—	18.1	2.01	2.08	7.1	5.7	6.98	3.47
ext. hallucis longus	EHL	20.9	24.3	7.48	9.4	1.25	—	19.4	1.22	1.26	4.07	2.74	4.01	3.28
flex. digitorum longus	FDL	20.3	27.3	4.46	13.6	1.16	—	23.6	1.06	1.12	5.94	4.53	5.77	5.47
flex. hallucis longus	FHL	38.9	26.9	5.27	16.9	1.62	—	16.6	2.06	2.24	10.1	7.21	9.68	4.71
gastrocnemius lateral	GL	62.2	22.4	5.88	12	2.24	1.41 [45]	9.96	3.95	4.13	9.06	9.92	8.86	2.24
gastrocnemius medial	GM	114	26.9	5.1	9.9	2.76	1.9 [45]	9.76	5.98	6.16	11.6	21.4	11.5	1.92
gluteus maximus	GMax	547	27	15.7	21.9	6.06	—	4.45	28.8	33.2	54.8	36	50.9	1.76
gluteus medius	GMed	274	20	7.33	20.5	4.97	—	4.02	19.4	22	32.9	36.1	30.8	1.58
gracilis	GR	52.5	28.7	22.8	8.2	1.82	—	15.8	2.6	2.65	6.39	2.22	6.32	2.43
iliacus	IL	114	20.6	10.7	14.3	3.16	—	6.52	7.84	8.33	14.7	10.2	14.3	1.82
peroneus brevis	PB	24.2	23.8	4.54	11.5	1.36	—	17.5	1.45	1.51	5.24	5	5.14	3.55
peroneus longus	PL	57.7	27.1	5.08	14.1	1.96	—	13.8	3.03	3.22	10.6	10.7	10.3	3.39
psoas	PS	97.7	24.3	11.7	10.6	2.7	—	8.98	5.72	5.92	11	7.83	10.8	1.89
rectus femoris	RF	111	36.3	7.59	13.9	2.35	1.69 [46]	15.5	4.33	4.59	16.6	13.9	16.1	3.72
sartorius	SA	78.5	44.8	40.3	1.3	1.78	—	25.2	2.49	2.49	2.87	1.9	2.86	1.15
semimembranosus	SM	134	29.3	6.9	15.1	2.88	—	10.2	6.5	6.97	18.4	19.1	17.7	2.73
semitendinosus	ST	99.7	29.7	19.3	12.9	2.47	2.33 [44]	12	4.77	5.02	13.6	4.92	13.3	2.79
soleus	SO	276	40.5	4.4	28.3	3.51	1.94 [48]	11.6	9.66	12.4	53.6	58.8	47.2	4.89
tibialis anterior	TA	80.1	26	6.83	9.6	2.36	2.46 [38]	11	4.38	4.5	9.12	11.1	8.99	2.05
tibialis posterior	TP	58.4	31	3.78	13.7	1.84	—	16.8	2.67	2.83	11	14.8	10.6	3.98
vastus intermedius	VI	172	41.2	9.93	4.5	2.75	2.05 [47]	15	5.93	5.96	9.14	16.8	9.11	1.54
vastus lateralis	VL	376	27.3	9.94	18.4	4.99	2.54 [31]	5.48	19.5	21.6	38.5	37	36.6	1.87
vastus medialis	VM	239	43.9	9.68	29.6	3.14	3.35 [47]	14	7.75	10.2	53.9	23.7	46.9	6.05

Powell *et al.*’s FCSA model endeavoured to capture the influence of muscle size, fibre length and pennation angle on force output. Yet, misinterpreting FCSA for PCSA suggests that muscle PCSA, as later commonly and properly conceived and depicted as running perpendicular to the main fibre direction, already includes a factor of the cosine of the pennation angle. This confusion of the muscle’s mechanical action (indeed measured in terms of force, i.e. FCSA) with the anatomical design parameter PCSA would imply that the greater the pennation angle, the lower the PCSA—a prediction that we know to be untrue. In fact, pennate design allows for a greater muscle PCSA within a given volume and thus *higher* forces per unit muscle belly mass as compared with a parallel arrangement of fibres [17,18].

A potential explanation for this misconception is that the maximum isometric forces measured by Powell *et al.* were characterized at ‘the muscle tendons (through the nylon ligature)’, which were then attached to an isometric transducer. Thus, it is perhaps not surprising that a model which aims to best fit muscle architecture parameters to muscle–tendon forces, should take into account the direction of the fibres. This is significant because when fibres are arranged at an angle relative to the primary axis of force generation (the pennation angle measured with respect to the tendon’s line of action), some of their force will not be transmitted along the tendon but perpendicular to it, i.e. off-axis. However, while focusing on the projection issue, it had been evidently ignored that arranging muscle fibres at an angle *at a given muscle volume* allows increasing the number of fibres acting in parallel, which over-compensates the loss of tendon force by diminishing projection. Resolving the mechanistic and functional consequences of this design strategy is the core aim of this work.

More recently, this seemingly ‘lost force’ (off-axis) has been shown to facilitate muscle shape changes and dynamic fibre rotations during active contractions [19,20]. This has certainly been another reason why PCSA (in fact, FCSA) has been used to represent the projection effect of pennation on the muscle fibres’ *in situ* force output *along* the tendon. Some have debated the functional significance of pennation angle, be it in terms of ‘force generation’ along the tendon, ‘lost force’ off-axis to the muscle’s primary line of action [21] or ‘force capacity’ as a morphological determinant [22].

**Figure 1.**
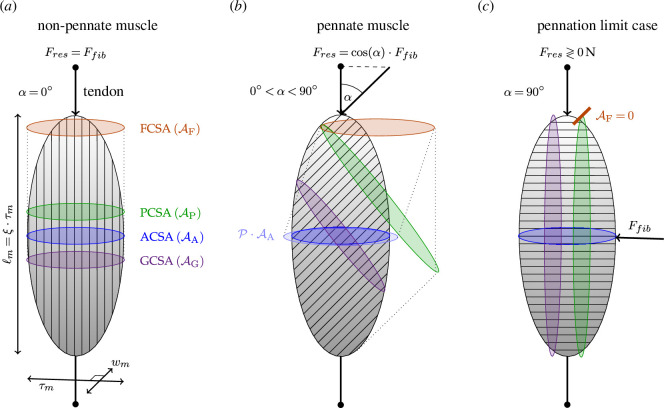
Simplified models of an ellipsoidal muscle with pennation angles $\alpha =0^{{}^{\circ}}$ (*a*), $0^{{\;}^{\circ }}<\alpha <90^{{\;}^{\circ }}$ (*b*) and the limit case $\alpha =90^{{}^{\circ}}$ (*c*). The blue, purple, green and bronze ellipses demonstrate ACSA (symbol $A_{\text{A}}$ ), geometric cross-sectional area (GCSA; symbol $A_{\text{G}}$ ), PCSA (symbol $A_{\text{P}}$ ) and FCSA (symbol $A_{\text{F}}$ ), respectively. Muscle dimensions are given by the pairwise perpendicular thickness ( $\tau_{m}$ ), width ( $w_{m}$ ) and length ( $\ell_{m}$ ), the latter expressed in terms of the length-to-thickness ratio ( ξ ). The directions of fibre force ( $F_{fib}$ ) and resulting (tendon) force in the line of muscle action ( $F_{res}$ ) are indicated by arrows. The light blue ellipse in (*b*) represents the pennation mechanical advantage (PMA), i.e. the enhancement of ACSA by the factor $P=A_{\text{F}}/A_{\text{A}}>1$ owing to the pennate arrangement of fibres. In this sketch, for ξ=2 and $\alpha =45^{{}^{\circ}}$ , the value of P≈1.11 (see equation (2.5)) corresponds to an increase in maximum force by approximately 11%.

One additional challenge is the ambiguous use of terminology and methodology across the literature. For example, contrary to equations (1.1) and (1.2), there remains confusion regarding how PCSA should be defined: by equating it with FCSA [10,23], by dividing the volume by fibre *rest* (instead of optimal) length [24, eqn 8], by dropping the condition of being perpendicular to fibre orientation [25, fig. 365B] or by being perpendicular to but not spanning all fibres within the muscle [26, fig. 1, right]. Also, there is disagreement whether the various CSAs are assumed to be unique, e.g. *anatomical* cross-sectional area (ACSA) to be the largest possible cut across the belly or whether they change with the location of the cut [27, §2.1] or with the current state of the muscle [24, table 1], respectively. As another example, in studies that use magnetic resonance imaging to characterize muscle architecture [28–30], ACSA is commonly termed FCSA. In summary, the mechanical advantage of pennate muscle designs, as well as the trade-off between increasing PCSA and an accompanying decrease in force by projection, owing to the pennate arrangement, are yet to be fully explored and interpreted.

Thus the aim of this study was to develop a three-dimensional geometric model of skeletal muscle that demonstrates the functional significance of pennation angle. To achieve this, we first create a range of conceptual models to demonstrate links between muscle size, shape and architecture. We then use our models to determine the direction and magnitude in which the potential force-producing benefits are offset by the pennate arrangement of fibres—as has previously been suggested [21]. We verified our model against gold standard, quantitatively measured architectural parameters for a range of skeletal muscles with differing fibre arrangements, shapes and sizes.

## 2. Various cross-sectional areas: distinction and calculation

We use one of the simplest three-dimensional models (figure 1), a muscle belly of ellipsoidal shape with length $\ell_{m}$ , thickness $\tau_{m}$ , width $w_{m}$ and thus volume $V_{m}=\ell_{m}\cdot \tau_{m}\cdot w_{m}\cdot \pi /6$ . To better distinguish between thickness and width, let the former ( $\tau_{m}$ ) denote the dimension perpendicular to $\ell_{m}$ with the *maximum* three-dimensional fibre pennation angle α and the latter ( $w_{m}$ ) the dimension perpendicular to both $\ell_{m}$ and $\tau_{m}$ . Hence, the so-called ACSA (symbol $A_{\text{A}}$ ), i.e. the largest cut through the belly perpendicular to the line of force action, constitutes an ellipse of area:


(2.1) $$A_{\text{A}}=\tau_{m}\cdot w_{m}\cdot \frac{\pi }{4}\;\;\;\;.$$

Note that—unlike most literature on PCSA [5,12,15,26,27,29,31–33]—we have introduced mathematical symbols when talking about the concrete values of the various CSAs ( A for ‘area’) to distinguish between the words and the variables. We advocate for adapting this strategy to avoid potential confusion; or at least use italic abbreviations, cf. [2,16,24]. Tendons are assumed to transfer their longitudinal contractile force perpendicular to ACSA in a straight line, without any re-direction around joints. Furthermore, we express the length of the muscle belly in terms of the thickness and the length-to-thickness ratio ξ :


(2.2) $$\ell_{m}=\xi \cdot \tau_{m}.$$

In non-pennate muscle ( $\alpha =0^{{}^{\circ}}$ ), fibres are assumed to be arranged in parallel to the line of (tendon) force action (the muscle output). The pennation angle hence is a measure of the deviation of the fibre direction from this line of action. While, per definition, ACSA remains the same regardless of pennation, other CSA definitions vary with α . At least, three further pennation-dependent CSAs should be distinguished (figure 1): (i) the *geometric* CSA (GCSA, symbol $A_{\text{G}}$ ) as a single cut through the thickest part of the muscle belly *perpendicular* to the fibre orientation; (ii) the *physiological* CSA, i.e. PCSA, as the conceived area, parallel to the GCSA, containing the orthogonal projection of *all* muscle fibres in parallel; and (iii) the *functional* CSA, i.e. FCSA, as the projection of the PCSA onto a plane parallel to ACSA, likewise *perpendicular* to the line of tendon force action.

Figure 1 illustrates different muscle architectures, with pennation angles α between $0^{{}^{\circ}}$ , where fibres run parallel to the line of tendon force action, and $90^{{}^{\circ}}$ , where fibres run perpendicular to the line of tendon force action, a very hypothetical limit case. In the first case $\alpha =0^{{}^{\circ}}$ (figure 1*a*), GCSA, PCSA and FCSA all coincide with the muscle belly’s ACSA. Here, fibre force $F_{fib}$ and tendon force output $F_{res}$ are equal. In the hypothetical limit case $\alpha =90^{{}^{\circ}}$ (figure 1*c*), ACSA still represents the base area, GCSA and PCSA coincide and obtain the value of $\ell_{m}\cdot \tau_{m}\cdot \pi /4$ and FCSA vanishes, i.e. the tendon force (output) is zero, as all fibres apply their forces perpendicular to the tendon line of action. Mathematically, FCSA becomes a line of length $w_{m}$ , the orthogonal projection of PCSA onto ACSA, with area zero (see the short bronze line in figure 1*c*, made slightly thicker for a better visibility). The notation $F_{res}≷0\;$ *N* thereby implies that other effects than contracting fibres may influence the resulting muscle force, see §5 and §6 for details.

**Figure 2.**
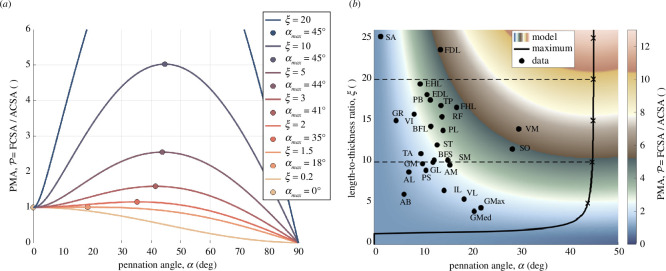
(*a*) PMA (symbol P ) as a function of pennation angle ( α ) at several length-to-thickness ratios ( ξ , coloured lines). Corresponding discrete maxima are marked with dots of the same colour. The value ξ=1.5 was chosen as close to the critical value $\xi =\sqrt{2}$ , at which the derivative dP/dα at $\alpha =0^{{}^{\circ}}$ changes its sign. (*b*) PMA landscape as a function of pennation angle and length-to-thickness ratio. Black crosses and dashed lines indicate landmarks for ξ being multiples of 5 and 10, respectively, on the continuous maximum ridge (black line). Muscles from Ward *et al.* [12] (black dots) are marked with abbreviations and values summarized in table 1. Most muscles show PMA values between 2 and 5, indicating that they can produce between two and five times the force than if they were non-pennate. The design of *vastus medialis* (VM) even suggests a sixfold increased maximum force owing to high pennation angle and considerable length-to-thickness ratio. For shapes other than ellipses, see the landscapes presented in figure 4.

The most interesting (non-trivial) cases are thus within $0^{{\;}^{\circ }}<\alpha <90^{{\;}^{\circ }}$ , an example is given in figure 1*b*. ACSA represents the externally visible belly shape and remains the same, for all values of α . The ellipsoidal GCSA can be calculated in closed form—as shown in detail in appendix A—being perpendicular to the fibre orientation, but not necessarily comprising all fibres within the belly. As a first approximation for small angles, it holds that $A_{\text{G}}\approx A_{\text{A}}/cos\left(\alpha \right)$ . In other words, ACSA would be the projection of GCSA onto the plane perpendicular to the tendon line of action. Intriguingly, nearly any sketch within the literature—e.g. [26, fig. 1, left], [33, fig. 2B] or [15, fig. 1]—depicts or implies that GCSA and PCSA are the same, presumably owing to the enticing parallelogram-shaped sketches, which even date back to anatomical drawings from the seventeenth century [9, tabula II]. We discuss the consequences of this interpretation below.

Indeed, PCSA lies parallel to GCSA, but by contrast, comprises all fibres within the muscle belly. PCSA can thus be displayed as another projection into the plane perpendicular to the fibre direction, exceeding the boundaries of the muscle belly (green plane in figure 1*b*). We are not aware of any sketch in the literature that demonstrates this fact. For an ellipsoidally shaped belly, PCSA can be calculated by


(2.3) $$A_{\text{P}}=A_{\text{A}}\cdot \sqrt{cos\;{\left(\alpha \right)}^{2}+\xi^{2}\cdot sin\;{\left(\alpha \right)}^{2}}\;,$$

which is equivalent to the calculation presented in equation (1.1), as shown in appendix A (after equation (A 5)). PCSA can further be projected onto the plane perpendicular to the line of tendon action to yield FCSA through


(2.4) $$A_{\text{F}}=A_{\text{P}}\cdot cos\;\left(\alpha \right)=A_{\text{A}}\cdot P\;,$$

which is in turn equivalent to the calculation presented in equation (1.2). The ratio of $A_{\text{F}}$ and $A_{\text{A}}$ indicates the factor by which the fibre arrangement functionally enhances the anatomically prescribed CSA, which we term here the *pennation mechanical advantage* (PMA; symbol P ):


(2.5) $$P=\frac{A_{\text{F}}}{A_{\text{A}}}=\sqrt{cos\;{\left(\alpha \right)}^{2}+\xi^{2}\cdot sin\;{\left(\alpha \right)}^{2}}\cdot cos\;\left(\alpha \right).$$

This factor illustrates (figure 1*b*) the extent to which pennation gears up the isometric force of the conceived non-pennate muscle with the very same muscle belly shape. Note that similar formulae as presented in equation (2.5) hold if differently shaped muscle bellies are considered (cf. again appendix A). In the special case of a rectangular prism (equation A 3), a cognate formula emerges in the literature from investigating angulated fibre attachment in ant mandibles [34, eqn 6].

We can predict the isometric tendon output force $F_{res}$ from given isometric stress σ measured perpendicular to the fibres, i.e. in the GCSA- or PCSA-plane (maximum value in the range 20–30 N cm^−2^ [35,36, p. 518], [37, p. 20]), the pennation angle α and the three ellipsoidal belly dimension parameters $\tau_{m}$ , $w_{m}$ and ξ : given the fibre force $F_{fib}=A_{\text{P}}\cdot \sigma$ , the tendon output force $F_{res}$ writes


(2.6) $$F_{res}=F_{fib}\cdot cos\;\left(\alpha \right)=A_{\text{F}}\cdot \sigma =A_{\text{A}}\cdot P\cdot \sigma \;.$$

## 3. Data

To demonstrate the values *P* of PMA across a range of human skeletal muscles with known functional differences, and to compare our model predictions to the traditional measures of PCSA and FCSA according to equations (1.1) and (1.2), respectively, we exploit an extensive dataset on lower extremity muscles [12, table 3]. These authors reported—among other parameters—muscle belly mass M , belly length $\ell_{m}$ , fibre length $\ell_{fib}$ , pennation angle $\alpha_{rest}$ and experimentally determined PCSA ${,}_{exp}$ . Muscle and fibre lengths had been scaled to an optimal sarcomere length of 2.7 μm and assumed to reflect a muscle during a maximum voluntary contraction (MVC). The pennation angle $\alpha_{rest}$ , however, was given in the resting position and not altered to reflect MVC. Several previous studies [15,38–43] have shown the potential for pennation at MVC to change substantially, but here the originally reported values were used. Note that the definition of ‘PCSA’ presented in Ward *et al.* [12] already included the $\cos(\alpha_{rest})$ factor to account for the pennation-induced decrease of force along the tendon and thus should have been rather described as FCSA—in accordance with Ward *et al.* having chosen the traditional approach through equations (1.1) and (1.2). Hence, we re-scaled their reported ‘PCSA’ values by $1/\cos(\alpha_{rest})$ to recover their experimentally and traditionally determined PCSA, i.e. $A_{\text{P},\exp}=M/\left(\rho \cdot \ell_{fib}\right)$ in accordance with equation (1.1) and ρ≈1.056 g/cm ${,}^{3}$ denoting the fibres’ mass density.

From the values given in Ward *et al.* [12, table 3], we first calculated an approximated $A_{\text{A}}=M/\left(\rho \cdot \ell_{m}\right)$ . Subsequently, assuming the muscle belly to be of circular base (i.e. $w_{m}=\tau_{m}$ ), we calculated width and thickness by $w_{m}=\tau_{m}=2\cdot \sqrt{A_{\text{A}}/\pi }$ . The resulting thickness was used to determine the length-to-thickness ratio ( $\xi =\ell_{m}/\tau_{m}$ , equation (2.2)), which led to the theoretically predicted PCSA ( $A_{\text{P}}=A_{\text{A}}\cdot \sqrt{cos\;{\left(\alpha_{rest}\right)}^{2}+\xi^{2}\cdot sin\;{\left(\alpha_{rest}\right)}^{2}}$ , equation (2.3)). Finally, the corresponding values of FCSA ( $A_{\text{F}}=cos\;\left(\alpha_{rest}\right)\cdot A_{\text{P}}$ , equation (2.4)) and PMA ( $P=A_{\text{F}}/A_{\text{A}}$ , equation (2.5)) were calculated. The given values $A_{\text{P},\exp}$ as well as thickness measurements of various muscles [31,38,44–47] served as a validation of the model predictions. All calculations were conducted in MatLab, v. 2023b (Mathworks, Natick, MA, USA).

## 4. Results

Figure 2*a* shows, for an ellipsoidally shaped muscle belly, the relationship between PMA, pennation angle α and the length-to-thickness ratio ξ for a range of muscle designs. From analysing the derivative of P(α) (see equation (A 6)), it follows that for muscles with $\xi \leq \sqrt{2}$ , i.e. being less than approximately 1.4 times longer than thick, PMA decreases strictly monotonically with α from P=1 at $\alpha =0^{{}^{\circ}}$ to P=0 at $\alpha =90^{{}^{\circ}}$ . Contrary, for muscles which are more than 1.4 times longer than thick ( $\xi >\sqrt{2}$ ), PMA initially increases up to a global maximum somewhere at pennation angles less than $45^{{}^{\circ}}$ (see appendix A for details), before eventually decreasing to zero, a result of the cos(α) term: only this component of a fibre’s pulling force projects onto the tendon direction. The larger ξ , the higher the P maximum and thus also the steeper the rise of P(α) with α , and the greater the angle $\alpha_{max}$ at which the maximum occurs (swiftly rising with ξ and saturating at $\alpha =45^{{}^{\circ}}$ ).

Figure 2*b* illustrates the corresponding heatmap of PMA as a function of length-to-thickness ratio ξ and pennation angle α . The ridge of PMA maxima ( $\alpha_{max}(\xi )$ , black line) is shown for ξ up to 26. Within this landscape, the designs of various muscles from table 1 are represented by black dots. The pennation angles of most muscles lie in the range between 8° and 17° (mean: 13.3°, s.d.: 6.28°, min: 1.3° for *sartorius*, SA, max: 29.6° for *vastus medialis*, VM) with nearly all muscles being far off the maximum ridge design, with four to five of them (*soleus*, SO; *vastus lateralis*, VL; VM; *gluteus maximus*, GMax; *gluteus medius*, GMed) tending towards it.

Comparing, through table 1, the predicted $A_{\text{P}}$ with the experimentally derived $A_{\text{P},\exp}$ , we see a generally good agreement. However, a significant (see figure 6*b*) systematic deviation $\left(\frac{A_{\text{P}}-A_{\text{P},\exp}}{A_{\text{P},\exp}}\right)$ exists, with a median of 22% (range between −46% for GMed and +187% for *gracilis*, GR). Theoretically, predicted thickness $\tau_{m}$ tendentially overestimated experimentally obtained literature values $\tau_{m,lit}$ with the deviation $\left(\frac{\tau_{m}-\tau_{m,lit}}{\tau_{m,lit}}\right)$ showing a median of 36% (range between −6% for VM and +96% for VL). Reasons for both of these overestimations are addressed in the discussion. The GCSA of all muscles expectedly lies between the values of ACSA and PCSA, but closer to the former. On average, GCSA was 7% larger than ACSA with a range of 0.5% (SA) and 32% (VM). Finally, the theoretically predicted PMA lies between 1.15 (SA) and 6.05 (VM) with a mean of 2.82 and s.d. of 1.30 across all lower limb muscles.

## 5. Discussion

The functional significance of pennate muscle design has far-reaching implications for the study of biomechanics, such as its shaping impact on the evolution of locomotion in humans and animals. Here, we developed physiologically informed geometric muscle models to demonstrate the mechanical implications of pennation angle. To clarify historical inconsistencies in the anatomical and mechanical interpretation of a muscle’s force-generating capacity owing to its physiological cross-sectional area (PCSA), we introduced the concept of pennation mechanical advantage (PMA). As a measure of force production per given muscle volume, PMA captures the impact of muscle design, in terms of a range of fibre architectures and belly shapes, on its mechanical output, and thus the functional implications of design across scales.

Skeletal muscle is the motor that powers human and animal locomotion. However, muscle size (i.e. volume) is not the best anatomical predictor of muscle force, but rather it is the number and orientation of muscle fibres [10]. This is perhaps not surprising given that (i) any muscle requires finite space which is generally limited in any region of the body, and (ii) any muscle volume requires metabolic energy for contractions, as well as maintenance [49,50]. Given these considerations, PMA allows transparent comparisons between muscles of a certain volume. It considers the potential trade-offs for necessary mechanical functions (e.g. force generation, high contraction speeds, optimized work or power generation; although speed issues have not been treated here) and their scaling across different body sizes. PMA solely depends on two design parameters and reveals the force advantage of moderately pennate muscles that are significantly longer than wide. That is, for a length-to-thickness ratio greater than $\sqrt{2}$ , and sufficiently small pennation angles, PMA exhibits a maximum value greater than 1, which implies that in a pennate muscle ( $\alpha >0^{{}^{\circ}}$ ) the functional share of PCSA (i.e. FCSA) is greater than what could have been achieved in a non-pennate muscle ( $\alpha =0^{{}^{\circ}}$ ) with the same ACSA and belly length. Our model predicts that most muscles in the human lower limb possess PMA values between 2 and 5, indicating that they can produce up to five times more force than if they were non-pennate. In fact, the muscles with the largest PMA (GMed, GMax, SO, VM and VL; see figure 2*b*) are the mono-articular extensors of each of the lower limb joints—ankle (SO), knee (VL and VM) and hip (GMax and GMed). These muscles are considered ‘antigravity’ muscles and produce low constant forces to enable upright posture as well as high transient forces during tasks such as walking, running and jumping.

Other functional interpretations of *in vivo* data can arise from our analysis. Anatomical studies, for example, the extensive dataset provided by Ward *et al*. [12], captures muscle architecture in passive, dissected muscle. However, ultrasound imaging of *in vivo* muscle contractions demonstrates that pennation angles can and do change substantially during fixed-end isometric contractions, see [40,45] and the electronic supplementary material, video. For example, pennation angles of the *gastrocnemius medialis* (GM) increased substantially, i.e. from 17° at rest to 35° at MVC [43, table 1]. Note that the terminology ‘isometric’ in this context refers to the whole muscle–tendon unit, but allows for the fibres within the belly to contract and stretch the elastic tendon and thus change length. Our model predicts that, under this assumption, rotation of fibres will result in an increased PMA or a rightward shift in the data points in figure 2*b* towards the maximal ridge. The calculations done herein including the resting angles may also have affected our prediction of thickness thus leading to an overestimation of literature values.

**Figure 5.**
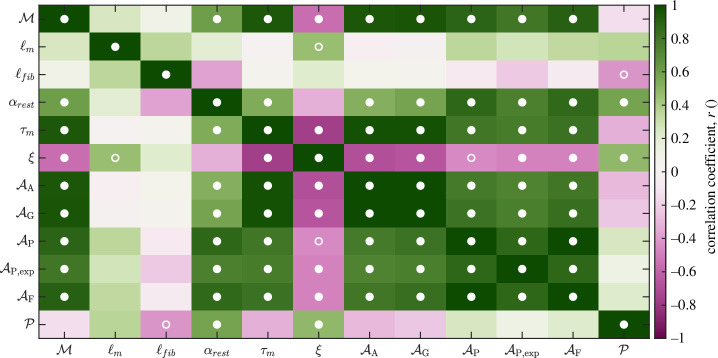
Correlation coefficients between each pair of optimized model parameters, see table 1. White closed and open circles indicate 99% and 95%, respectively, significance levels for the deviation of the correlation coefficient r from zero. The test statistics were calculated as $t=\frac{r.\sqrt{n-2}}{\sqrt{1-\rho^{2}}}$ [68] and compared against the corresponding quantile of the Student’s t -distribution with n−2 d.f. (two-sided test), where n=28 is the number of available datasets.

**Figure 6.**
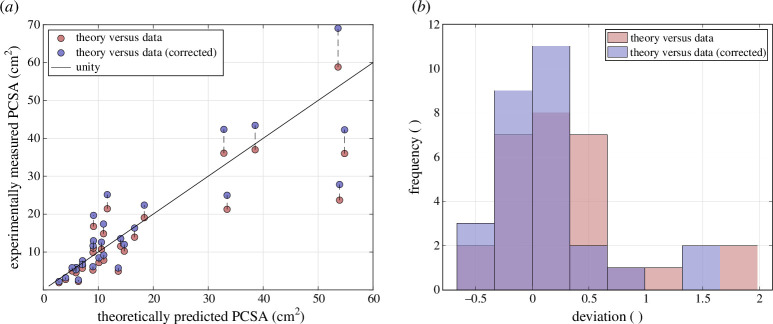
(*a*) Theoretically predicted versus experimentally measured PCSA values as presented in table 1 (red dots) and after correction by the factor 2.7/2.3 (blue dots). The latter are supposed to better represent human sarcomeres of optimal fibre length at 2.3 μm instead of 2.7 μm (cf. [12, eqn 1]). The coefficient of determination when using unity (black line) as the assumed relation rises from 0.63 to 0.74 upon correction. (*b*) Deviation $\left(\frac{A_{\text{P}}-A_{\text{P},\exp}}{A_{\text{P},\exp}}\right)$ between theory and experiment before (red) and after (blue) correction. A *t*-test reveals that the deviation between the theory and the uncorrected data is significantly different from zero ( p≈0.0036 ), while the deviation between the theory and the corrected data is not significant ( p≈0.11 ).

Muscles change length and produce force to generate the work and power required for locomotor tasks. Our model establishes that a muscle’s maximum force production is enhanced by moderate pennation angles. Indeed, this is correlated with increased PCSA values, however, our new parameter PMA reveals the mechanical advantage of pennate muscle design: the ratio of two CSA measures, $A_{\text{F}}$ and $A_{\text{A}}$ , rather than $A_{\text{P}}$ . Our models’ predictions of $A_{\text{P}}$ (equation (2.3)) are largely consistent with experimental measurements of PCSA from lower limb muscle dissections ( r≈0.86 , see figure 5 in appendix B). The deviation between our theoretical prediction and the experimentally determined $A_{\text{P},\exp}$ , accounted for a median of approx. 22%. We hypothesized that this systematic deviation in $A_{\text{P}}$ could well be a result of the normalization in fibre lengths by Ward *et al*. [12] to an optimal sarcomere length value of 2.7 μm, a value at the upper bound of human optimal sarcomere length, whereas it is more typically reported in the range between 2.3 and 2.5 μm [51]. When $A_{\text{P},\exp}$ was normalized to 2.3 μm, then the median deviation only accounted for 2% (range −54% to +145%) with no significant difference from the theoretical prediction, see figure 6 in appendix B. The coefficient of determination ( $R^{2}$ ) also improved upon refined normalization from 0.63 to 0.74. If the optimal sarcomere length was treated as a free parameter, a maximum of $R^{2}=0.75$ could be found at around a still reasonable 2.17 μm. Note that, while the optimal length of sarcomeres or single fibres can be determined using *ex vivo* or invasive approaches, the ability to measure (average) optimal fibre length in whole muscle bellies *in vivo* is experimentally challenging [6]. For a reliable estimate of optimal fibre length, it might be necessary to collect measurements of fibre length during maximum isometric contractions under different muscle lengths (or *in vivo* joint configurations) [52] and ideally at different regions of the muscle belly.

We provide a geometric muscle model, informed with empirical data, that demonstrates the functional importance of pennate muscle architectures on PCSA and provides a new parameter, PMA, to characterize this mechanical advantage. So, when should we use PCSA and when should we opt for PMA? If one’s goal is to estimate a muscle’s maximum force-generating capacity, for example, as input to a Hill-type muscle model, we suggest estimating PCSA using equation (2.3). Yet we acknowledge that, in practice, measuring muscle dimensions (thickness and width) *in vivo* remains an experimental challenge. Our goal is not to disregard the use of equation (1.1) for determining PCSA ( =volume/fibre length ), but rather to highlight (as others have [21]) that pennation angle should only be used to scale between fibre forces and tendon forces but not within equation (1.1). Conversely, if one’s purpose is to compare the functional implications of different muscle fibre architectures across muscles, individuals, species or scales—we suggest adopting PMA, which captures the influence of muscle design on mechanical performance.

## 6. Conclusion and outlook

We have investigated the effect of pennation angle as a design parameter on a muscle’s force-generating capacity. Our results demonstrate the functional significance of pennation angle on this mechanical output during isometric MVCs with likely implications for locomotor function during more dynamic tasks. Therefore, we have captured a variety of three-dimensional geometries that constitute a range of form-simplifying, yet anatomically realistic architectures. These approximate the geometric shapes and a basic mechanical condition of skeletal muscle *in vivo*. It is well established that muscle geometry has a considerable influence on three-dimensional contractile dynamics, e.g. cylindrical versus ellipsoidal muscle bellies during transversal loading [53]. Furthermore, when muscles contract, they bulge radially (albeit, in general, not equally in all directions [53]) to maintain constant volume [54] and fibres rotate to steeper pennation angles [45,55]. These shape changes and fibre rotations induce variable ratios between whole muscle belly and single fibre length changes—a quantity known as gearing [19,20]. Experimental and modelling [3,19,20,56,57] studies across a range of animals and humans have illustrated that gearing boosts the mechanical performance of skeletal muscle by allowing muscle fibres to operate at more favourable conditions for force and power production over an extended length range. Skeletal muscles with greater pennation angles [20] and larger PCSAs [45] operate at higher gearing, suggesting that there is a dynamic advantage of pennation.

Our model can serve as a basis for future developments and enhancements. For example, combining two uni-pennate models into a single bi-pennate model will be possible by formulating the corresponding force (and torque) constraints as additional equations. This way, even the complex architecture of multi-pennate muscle designs [58] can be captured. Furthermore, the mechanical interaction of the muscle with the surrounding tissue may be modelled by deformation-(viscosity-)force laws in order to investigate the effects of transverse loading [59], extramuscular myofascial force transmission [60] or compressive forces [61] on muscle. Concomitantly, modelling the exact geometry of fibre attachment points on the bone (see the mentioned example regarding ant mandibles [22,34]), as well as muscles’ complex routing (e.g. wrapping) [62], may reveal their (potentially) substantial impact on the mechanical advantage of pennation.

Gradually extending our basic mechano-geometric model will also enable a deeper understanding of evolutionary trends, given that not all muscles are designed to generate high forces. Equipping pennate designs with a Hill-type force–velocity relation [63] might yield different optima when striving for maximum contraction velocity or maximum range of motion. Likewise, different fibre compositions (fast versus slow) [64] may be distinguished to identify designs striving for maximum work or power output [65,66]. There are many open questions surrounding pennate muscle designs. A careful definition of involved concepts such as PCSA—and a deductive succession of mechanistic models in reciprocity with reliable data—will advance our understanding of muscle form and function.

## Figures and Tables

**Figure 3.**
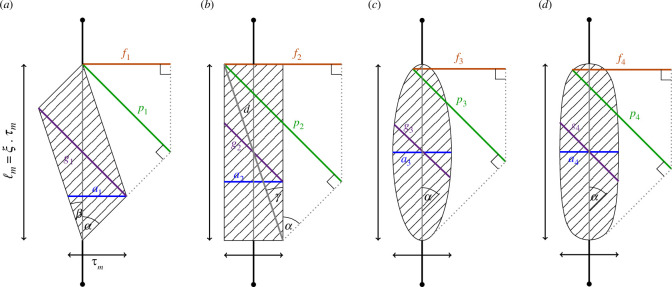
Four different possible shapes for a prismatic muscle geometry. From left to right: parallelogram (rhomboid), rectangle, ellipse and squircle (rectipse). All muscle bellies have the same length ( $\ell_{m}$ ), thickness ( $\tau_{m}$ ) and pennation angle ( α ). One-dimensional representations of various CSAs are shown in the corresponding colours (i.e. blue $a_{i}$ (ACSA), purple $g_{i}$ (GCSA), green $p_{i}$ (PCSA) and bronze $f_{i}$ (FCSA) for i=1...4 ), cf. figure 1. The PMA landscape for each shape, depending on length-to-thickness ratio ξ and pennation angle, is displayed in figure 4. For further explanation and calculation, see text.

**Figure 4.**
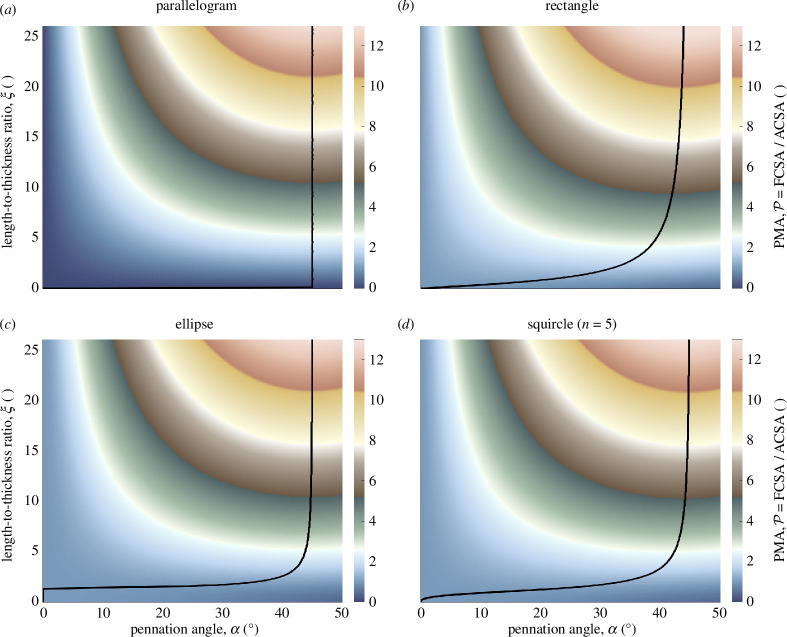
PMA ( P ) landscape as a function of pennation angle ( α ) and length-to-thickness ratio ( ξ ) for the various muscle belly shapes shown in figure 3: (*a*) parallelogram with $P_{P}=\xi \cdot sin\;\left(\alpha \right)\cdot cos\;\left(\alpha \right)$ , (*b*) rectangle with $P_{R}=\left(cos\;\left(\alpha \right)+\xi \cdot sin\left(\alpha \right)\right)\cdot cos\;\left(\alpha \right)$ , (*c*) ellipse with $P_{E}=\sqrt{cos\;{\left(\alpha \right)}^{2}+\xi^{2}\cdot sin\;{\left(\alpha \right)}^{2}}\cdot cos\;\left(\alpha \right)$ , i.e. the same as presented in figure 2*b* and (*d*) squircle (rectipse) with $P_{S}={\left(cos\;{\left(\alpha \right)}^{n}+\xi^{n}\cdot sin\;{\left(\alpha \right)}^{n}\right)}^{1/n}\cdot cos\;\left(\alpha \right)$ with n=5 . For the derivation of the formulae, see text and equations in appendix A.

## Data Availability

All data and code used to create the figures are made available as the electronic supplementary material [67].

## Appendix A. Calculation of various cross-sectional areas for differently shaped muscles

Here, we address two issues. The first is to conflate the two definitions of PCSA, once through ‘volume divided by (average) optimal fibre length’ and once through ‘the projection of the entirety of fibres onto a plane perpendicular to them’. Second, this is done by presenting the various geometric shapes of muscle to reveal underlying connections. To facilitate calculations, we start with assuming the muscle to be of prismatic shape and that the width of the muscle belly (cf. figure 1) equals unity length, i.e. $w_{m}=1$ . Furthermore, any fibre is assumed to run within exactly one plane, perpendicular to the width direction. That is, a well-defined two-dimensional geometric body is extended into the third dimension by unity parameter $w_{m}$ . With this constraint, we ensure that the PMA of the prisms can directly be equated to the PMA of more general three-dimensional shapes, as shown below.

We investigate a total of four basal-area shapes, each of the same thickness $\tau_{m}$ , same length $\ell_{m}=\xi \cdot \tau_{m}$ and same pennation angle α : (i) a parallelogram (more exactly, a rhomboid); (ii) a rectangle; (iii) an ellipse; and (iv) a *squircle*, refer figures 3 and 4. The latter is a special type of superellipse, being an established portemonnaie word from ‘square’ and ‘circle’, although it rather constitutes a transition between ellipse and rectangle (we propose it to be named ‘rectipse’). The CSAs of interest are represented by small letter equivalents of dividing the corresponding CSA by $w_{m}$ , i.e. $a_{i}$ (ACSA), $g_{i}$ (GCSA), $p_{i}$ (PCSA) and $f_{i}$ (FCSA) for i=1...4 . The connection of these quantities to the area of the four shapes ( $A_{P},A_{R},A_{E},A_{S}$ ) and the mean fibre length ( $\ell_{fib}$ ) is given. For the sake of clarity, all formulae are written to hold for $0^{{}^{\circ}}\leq \alpha \leq 90^{{}^{\circ}}$ only.

For the parallelogram, note that $g_{1}$ and $p_{1}$ are intentionally of the same length, which however constitutes a special case, as can be seen from the calculations. For those, we introduce an auxiliary angle β , which depends on $\ell_{m}$ , $\ell_{fib}$ and α through:

$$\beta =\text{atan}\left(\frac{\ell_{fib}\cdot sin\;\left(\alpha \right)}{\ell_{m}-\ell_{fib}\cdot cos\;\left(\alpha \right)}\right)\;.$$

For the derivation of this formula, consider the height of the triangle with base $\ell_{m}$ , which can be obtained by either $\ell_{fib}\cdot sin\;\left(\alpha \right)$ or by $tan\;\left(\beta \right)\cdot \left(\ell_{m}-\ell_{fib}\cdot cos\;\left(\alpha \right)\right).$ The cross sections follow directly as:

(A 1)
$$a_{1}=\tau_{m},\;\;\;\;g_{1}=a_{1}\cdot \frac{cos\;\left(\beta \right)}{cos\;\left(\alpha +\beta \right)}\;,\;\;\;\;p_{1}=\ell_{m}\cdot sin\;\left(\alpha \right),\;\;\;\;f_{1}=p_{1}\cdot cos\;\left(\alpha \right)=\ell_{m}\cdot cos\;\left(\alpha \right)\cdot sin\;\left(\alpha \right),$$

where the calculation of $g_{1}$ used the law of sines. The area of the parallelogram is given by $A_{P}=\ell_{fib}\cdot p_{1}=\ell_{fib}\cdot \ell_{m}\cdot sin\;\left(\alpha \right)$ , where $\ell_{fib}$ is connected to $a_{1}$ and $g_{1}$ through $a_{1}=\ell_{fib}\cdot \left(cos\;\left(\alpha \right)\cdot tan\;\left(\beta \right)+sin\;\left(\alpha \right)\right)$ or $g_{1}=\ell_{fib}\cdot tan\;\left(\alpha +\beta \right)$ , respectively. A directly visible disadvantage of the parallelogram shape is the vanishing value for $p_{1}$ as α approaches zero.

For the rectangle with area $A_{R}=\ell_{m}\cdot \tau_{m}$ , calculating $a_{2}$ and $g_{2}$ proves less complicated, while for the calculation of $p_{2}$ again an auxiliary angle γ between the diagonal of length d and the line of action has to be defined. The diagonal is of length $d=\sqrt{1+\xi^{2}}\cdot \tau_{m}$ owing to the Pythagoras’ theorem. The angle to the line of force action can be determined by

$$\gamma =\text{atan}\;\left(\frac{\tau_{m}}{\ell_{m}}\right)=\text{atan}\;\left(\frac{1}{\xi }\right)\;.$$

As $p_{2}$ is the projection of the diagonal with length d orthogonal to the fibre orientation, it follows immediately:

(A 2)
$$p_{2}=d\cdot sin\left(\alpha +\gamma \right)=\sqrt{1+\xi^{2}}\cdot sin\;\left(\alpha +\text{atan}\;\left(\frac{1}{\xi }\right)\right)\;.$$

Applying the addition theorem sin(α+γ)=cos(γ)⋅sin(α)+cos(α)⋅sin(γ) and using the relations $\sin(\text{atan}(1/\xi ))=1/\sqrt{1+\xi^{2}}$ as well as $cos\;\left(\text{atan}\;\left(1/\xi \right)\right)=1/\sqrt{1+\xi^{-2}}$ for ξ>0 yields a more compact representation:

(A 3)
$$a_{2}=\tau_{m},\;\;\;\;g_{2}=\frac{\tau_{m}}{cos\;\left(\alpha \right)},\;\;\;\;p_{2}=\tau_{m}\cdot \left(cos\;\left(\alpha \right)+\xi \cdot sin\;\left(\alpha \right)\right),\;\;\;\;f_{2}=\tau_{m}\cdot \left(cos\;\left(\alpha \right)+\xi \cdot sin\;\left(\alpha \right)\right)\cdot cos\;\left(\alpha \right).$$

The mean fibre length can be calculated by:

$$\ell_{fib}=\frac{A_{R}}{p_{2}}=\frac{\ell_{m}\cdot \tau_{m}}{\tau_{m}\cdot cos\;\left(\alpha \right)+\ell_{m}\cdot sin\;\left(\alpha \right)}\;.$$

Contrary to $p_{1}$ , the value of $p_{2}$ does not vanish for α→0 . Indeed, as shown in figure 2, $P=f_{2}/a_{2}=1$ for $\alpha =0^{{}^{\circ}}$ and P=0 for $\alpha =90^{{}^{\circ}}$ . In between, PMA exhibits a global maximum at

(A 4)
$$\begin{array}{l} (\alpha_{\max},P_{\max})=\left(\underset{\overset{\xi \to \infty }{\to }45^{\circ }}{\underbrace{45^{\circ }-\frac{1}{2}\cdot atan\left(\frac{1}{\xi }\right)}}\;,\;\frac{1}{2}\cdot \left(1+\sqrt{1+\xi^{2}}\right)\right) \end{array}$$

For the ellipse, let its centre be the origin of a coordinate system and the major semi-axes of length $\ell_{m}/2$ and $\tau_{m}/2$ correspond to the ordinate and abscissa direction, respectively. One possible representation in Cartesian coordinates reads as:

(A 5)
$$E=\left\{\left(\begin{array}{c} x\\ y \end{array}\right)\;|\;\frac{x^{2}}{{\left(\tau_{m}/2\right)}^{2}}+\frac{y^{2}}{{\left(\ell_{m}/2\right)}^{2}}\leq 1\right\}\;.$$

Calculation of the area $A_{E}=\ell_{m}\cdot \tau_{m}\cdot \pi /4$ and the value $a_{3}=\tau_{m}$ are straightforward. However, to calculate $g_{3}$ , the two intersection points of the line y=−tan(α)⋅x with the boundary of E and their distances have to be calculated. Inserting the line equation into equation (A 5), solving for x , inserting this value into the line equation to solve for y and finally calculating the norm yields:

$$g_{3}=\frac{\ell_{m}\cdot \tau_{m}\cdot sec\;\left(\alpha \right)}{2\cdot \sqrt{{\left(\tau_{m}/2\right)}^{2}\cdot tan\;{\left(\alpha \right)}^{2}+{\left(\ell_{m}/2\right)}^{2}}}\;.$$

The value of $p_{3}$ can be calculated by determining the distances between the two tangents with slope tan(α) , which can be represented by $y_{\pm }=tan\;\left(\alpha \right)\cdot x\pm \sqrt{tan\;{\left(\alpha \right)}^{2}\cdot {\left(\tau_{m}/2\right)}^{2}+{\left(\ell_{m}/2\right)}^{2}}$ . Calculating this distance between $y_{+}$ and $y_{-}$ yields:

$$\begin{array}{ll} p_{3} & =2\cdot \sqrt{\frac{tan\;(\alpha {)}^{2}\cdot (\tau_{m}/2{)}^{2}+(\ell_{m}/2{)}^{2}}{tan\;(\alpha {)}^{2}+1}}=\sqrt{\tau_{m}^{2}\cdot cos\;(\alpha {)}^{2}+\ell_{m}^{2}\cdot sin\;(\alpha {)}^{2}}\\ & =a_{3}\cdot \sqrt{cos\;(\alpha {)}^{2}+\xi^{2}\cdot sin\;(\alpha {)}^{2}}\;. \end{array}$$

Accordingly, $f_{3}=p_{3}\cdot cos\;\left(\alpha \right)$ and $\ell_{fib}=A_{E}/p_{3}$ . Note that the ratio $P=f_{3}/a_{3}=A_{\text{F}}/A_{\text{A}}$ is the same for an elliptical prism as it is for an ellipsoid, as the width $w_{m}$ of the FCSA- and the ACSA-ellipse is the same owing to its definition of being perpendicular to fibre pennation direction. In formulas, the areas of the elliptical prism $A_{\text{F}}=f_{3}\cdot w_{m}$ and $A_{\text{A}}=a_{3}\cdot w_{m}$ have the same quotient as the ellipse areas $A_{\text{F}}=f_{3}\cdot w_{m}\cdot \pi /4$ and $A_{\text{A}}=a_{3}\cdot w_{m}\cdot \pi /4$ . Furthermore, from calculating the derivative of P with respect to α at $\alpha =0^{{}^{\circ}}$ , i.e.

(A 6)
$$\frac{\text{d}P}{\text{d}\alpha }{|}_{\alpha =0^{{\;}^{\circ }}}=\frac{\left(\xi^{2}-2\right)\cdot sin{\left(\alpha \right)}^{2}\cdot cos\left(\alpha \right)-\xi^{2}\cdot sin{\left(\alpha \right)}^{3}}{\sqrt{\xi^{2}\cdot sin{\left(\alpha \right)}^{2}+cos{\left(\alpha \right)}^{2}}}{|}_{\alpha =0^{{\;}^{\circ }}}=\xi^{2}-2\;,$$

it follows that the slope of P(α) is greater than zero for $\xi >\sqrt{2}$ and smaller than zero for $\xi <\sqrt{2}$ . It even follows from further investigation that for $\xi <\sqrt{2}$ , the value P=1 for $\alpha =0^{{}^{\circ}}$ is a global maximum.

For the squircle (rectipse), a similar formula as equation (A 5) holds, using a natural number greater than or equal to two as exponent:

(A 7)
$$S=\left\{\left(\begin{array}{c} x\\ y \end{array}\right)\;|\;{\left|\frac{x}{\tau_{m}/2}\right|}^{n}+{\left|\frac{y}{\ell_{m}/2}\right|}^{n}\leq 1\;,\;n\in N_{\geq 2}\right\}\;.$$

In the case n=2 , it holds S=E and for n→∞ , it holds S→R . The area $A_{S}$ can be expressed in terms of the gamma function Γ :

$$A_{S}=\ell_{m}\cdot \tau_{m}\cdot \frac{\Gamma \;{\left(1+\frac{1}{n}\right)}^{2}}{\Gamma \;\left(1+\frac{2}{n}\right)}\;.$$

The calculation of $a_{4},g_{4},p_{4}$ follows the steps for $a_{3},g_{3},p_{3}$ and yields

$$a_{4}=\tau_{m}\;,\;\;\;\;g_{4}=\frac{\ell_{m}\cdot \tau_{m}\cdot sec\;\left(\alpha \right)}{2\cdot {\left({\left(\tau_{m}/2\right)}^{n}\cdot tan\;{\left(\alpha \right)}^{n}+{\left(\ell_{m}/2\right)}^{n}\right)}^{1/n}}\;,\;\;\;\;p_{4}={\left(\tau_{m}^{n}\cdot cos\;{\left(\alpha \right)}^{n}+\ell_{m}^{n}\cdot sin\;{\left(\alpha \right)}^{n}\right)}^{1/n}\;.$$

Accordingly, $f_{4}=p_{4}\;\cdot cos\;(\alpha )\;and\;\ell_{fib}=A_{s}/p_{4}\;.$
